# The Incidence and Clinical Burden of Respiratory Syncytial Virus Disease Identified through Hospital Outpatient Presentations in Kenyan Children

**DOI:** 10.1371/journal.pone.0052520

**Published:** 2012-12-26

**Authors:** Emelda A. Okiro, Mwanajuma Ngama, Ann Bett, D. James Nokes

**Affiliations:** 1 Malaria Public Health Department, KEMRI-Wellcome Trust Collaborative Research Programme, Nairobi, Kenya; 2 Centre for Tropical Medicine, Nuffield Department of Medicine, University of Oxford, Oxford, United Kingdom; 3 Centre for Geographic Medicine Research – Coast, Kenya Medical Research Institute, Kilifi, Kenya; 4 School of Life Sciences, University of Warwick, Coventry, United Kingdom; University of Hong Kong, Hong Kong

## Abstract

**Background:**

There is little information that describe the burden of respiratory syncytial virus (RSV) associated disease in the tropical African outpatient setting.

**Methods:**

We studied a systematic sample of children aged <5 years presenting to a rural district hospital in Kenya with acute respiratory infection (ARI) between May 2002 and April 2004. We collected clinical data and screened nasal wash samples for RSV antigen by immunofluorescence. We used a linked demographic surveillance system to estimate disease incidence.

**Results:**

Among 2143 children tested, 166 (8%) were RSV positive (6% among children with upper respiratory tract infection and 12% among children with lower respiratory tract infection (LRTI). RSV was more likely in LRTI than URTI (p<0.001). 51% of RSV cases were aged 1 year or over. RSV cases represented 3.4% of hospital outpatient presentations. Relative to RSV negative cases, RSV positive cases were more likely to have crackles (RR = 1.63; 95% CI 1.34–1.97), nasal flaring (RR = 2.66; 95% CI 1.40–5.04), in-drawing (RR = 2.24; 95% CI 1.47–3.40), fast breathing for age (RR = 1.34; 95% CI 1.03–1.75) and fever (RR = 1.54; 95% CI 1.33–1.80). The estimated incidence of RSV-ARI and RSV-LRTI, per 100,000 child years, among those aged <5 years was 767 and 283, respectively.

**Conclusion:**

The burden of childhood RSV-associated URTI and LRTI presenting to outpatients in this setting is considerable. The clinical features of cases associated with an RSV infection were more severe than cases without an RSV diagnosis.

## Introduction

In the developing world pneumonia represents the major public health disease burden [1], [2], [3]. Respiratory syncytial virus (RSV) is the main viral cause of severe pneumonia and bronchiolitis in infants and young children contributing considerably to the burden on health services [4], [5], [6]. Accordingly, the development of a vaccine for RSV is viewed as a priority [7], [8], [9].

Most of the information that is available on the clinical characteristics and epidemiology of RSV is derived from studies of paediatric patients admitted to hospital with severe respiratory illness. These data do not present the full extent of the health burden in the community since they exclude children with disease not severe enough to require hospital admission, which nonetheless has social, morbid and economic costs [10]. Such data are important in support of estimates of the effectiveness and costs associated with vaccine intervention.

This gap in our understanding of the RSV disease burden could be filled using data from outpatient facilities. However, few studies of RSV have been undertaken in outpatients [11], [12], and fewer still in the developing world [13]. Thus the burden of RSV-acute respiratory infections (RSV-ARI) in outpatient presentations in developing countries remains largely un-quantified [10], [14], and this increases the inaccuracy of assessment of its public health importance.

To fill this gap we undertook continuous surveillance for RSV disease in children who presented with acute respiratory infection (ARI) to Kilifi District Hospital (KDH) over a two-year period. Patients were linked to the population register of the Kilifi Health and Demographic Surveillance System (KHDSS) to estimate incidence. This study took place within the context of other studies on RSV in the same area [10], [14], [15], [16], [17], [18], [19], [20], [21], [22], [23].

## Methods

### Ethics Statement

Ethical clearance for this study was obtained from the Kenyan National Ethics Review Board and from Coventry Research Ethics Committee, UK and signed consent was obtained from the parent/guardian of every child in the study.

The study was undertaken at the outpatient department of KDH. The hospital is situated in Kilifi Town in Kilifi District a rural coastal town in Kenya, and has a major outpatient facility with around 15,000 paediatric presentations annually. Kilifi District covers an area of 4,779 km^2^ with a density of 114 persons/km^2^ and experiences a tropical climate with seasonal rainfall (March to July and October to December). Malaria is endemic with seasonal transmission following the rains [24]. In 2003, the projected district population was 614,106 [25]. Farmers dependent on agriculture make up 80% of the district population [26] and are largely of the Mijikenda ethic origin. The population had a growth rate of 3.1% per annum in 1999 with 18% under the age of 5 years [25].

Kilifi Health and Demographic Surveillance System (KHDSS) is a system of continuous surveillance for vital events and migration in an area of 891 km^2^ immediately surrounding Kilifi District Hospital. It was established in 2000 and is maintained by household visits approximately 3 times per year. In order to accurately estimate the incidence of ARI and of these the RSV cases, from May 2003 we determined the residency status within the KHDSS of all children recruited into the present study. Each outpatient record from the study population was matched to an enumeration record from the census to identify residency status at the time of recruitment. On this basis children were classified as either resident or non-resident within the KHDSS.

The study was carried out between the first week of May 2002 and the last week of April 2004. The KEMRI Wellcome Trust Research Programme manages a research clinic adjacent to the KDH outpatient department, with well-trained clinical officers and supported by haematology and virology laboratories. Each weekday morning a trained study field worker (FW) interviewed mothers presenting with children to the outpatient department of KDH. Children aged <5 years who had clinical signs of ARI were eligible for inclusion in the study. The FW recruited children in order of attendance aiming to meet a weekly target defined as the number of inpatient ARI admissions from the preceding week. ARI was defined as at least one of: (i) runny/blocked nose; (ii) cough and fever; (iii) difficulty in breathing or; (iv) cough with fast breathing.

Before each child was seen by a study clinical officer (CO) details on location of residence were first determined following which measurements were made of oxygen saturation (pulse oximetry, Nellcor®), weight, heart and respiratory rates, and axillary temperature on a standard proforma. Each child was then examined by a study CO who recorded the clinical history and examination on the standard proforma and made the decision to request for a nasal wash sample. Among children with an axillary temperature ≥37.5°C, or with a history of fever in the previous week, a blood slide was taken to diagnose malaria (by Giemsa staining). Nasal wash specimens were examined for RSV antigen using a direct immunofluorescence test (DFA; Chemicon). Details of specimen collection and laboratory processing are reported elsewhere [14].

We categorized ARI cases into lower respiratory tract infections (LRTI) and upper respiratory tract infections (URTI). LRTI was defined as acute cough or difficulty in breathing in association with one or more of the following: (1) increased breathing rate for age, (2) lower chest wall indrawing, or (3) inability to feed or reduced consciousness or level or hypoxia (O_2_ saturation <90%), with severe LRTI defined as either 2 or 3 as previously described [14]. URTI was defined as acute cough or difficulty in breathing or nasal congestion/discharge, without further signs of severe respiratory disease.

The target population was the total population of children less than 5 years presenting to KDH outpatient department with ARI during the study period. To characterize the target population, we extracted all the information of visits made during the study period from KDH outpatient registers. In this population a clinical diagnosis was ascribed by the Government clinician and this was recorded in the patient registers. However, information on patient specific symptoms is not routinely recorded in the outpatient register and is usually recorded in the patient record books which are retained by the guardians and were therefore not available. Thus diagnoses among children who were not included in the RSV study were made without reference to the study criteria and we relied on the diagnoses of URTI or LRTI made by the clinicians in the KDH outpatient department. We defined the target population as the total population presenting to KDH outpatient department with a respiratory infection and those selected for the study are referred to as the study population.

The Meteorological data used in this study was collected at the Kilifi Agricultural Institute located less than 2 kilometers away from KDH. Daily data on rainfall, relative humidity and maximum and minimum temperatures was available for the whole study period. For each month we computed the total monthly precipitation (mm), relative humidity (RH), and average maximum and minimum temperatures.

### Data Analysis

Statistical analysis was undertaken using Stata version 11.0 (StataCorp LP, TX USA) and Microsoft Excel 2007. The pattern of presentations to hospital with RSV-associated infection or disease was determined among children presenting between May 2002 and April 2004. Because residency status within KHDSS was only obtained from May 2003 onwards we restricted the analysis of incidence to May 2003–April 2004. A comparison of age, sex, and severity distributions of the study sample with the target population was done using chi square tests.

The following three factors have a possible bearing on incidence estimation in this study. First, residency status within KHDSS is not routinely recorded for outpatient department presentations. Second, LRTI definitions used by the outpatient department were not those applied to the study sample. Third, the study sample differed from the outpatient department in the proportion of infants. To account for these factors, the number of ARI presentations to the outpatient department was scaled by the proportion of the study sample identified as KHDSS resident (66.18%), the number of LRTI presentations to the outpatient department was estimated as the number of KHDSS resident ARI cases scaled by the proportion of children in the study population diagnosed as LRTI, and the scaling was applied separately for infants and children 1–4 years of age. In so doing we are assuming that (i) the residency prevalence and (ii) the study-defined LRTI prevalence in the target population was the same as for our study population.

Incidence of ARI and LRTI in age group *i* per 100,000 population per year was estimated from the number of KHDSS resident cases presenting to the government outpatient department in 2003/04, and the mid-year (2003/04) KHDSS population, for age group i = <12 months, 1–4 years and <5 years. The resident population of KHDSS in the age-strata 0–11 months and 1–4 years was estimated to be 8123 and 31 870, respectively, on 30^th^ October 2003. The incidence of RSV-LRTI cases was defined as the incidence of LRTI multiplied by the proportion of the study sample that was RSV positive.

## Results

### Population Characteristics

**Figure 1:**
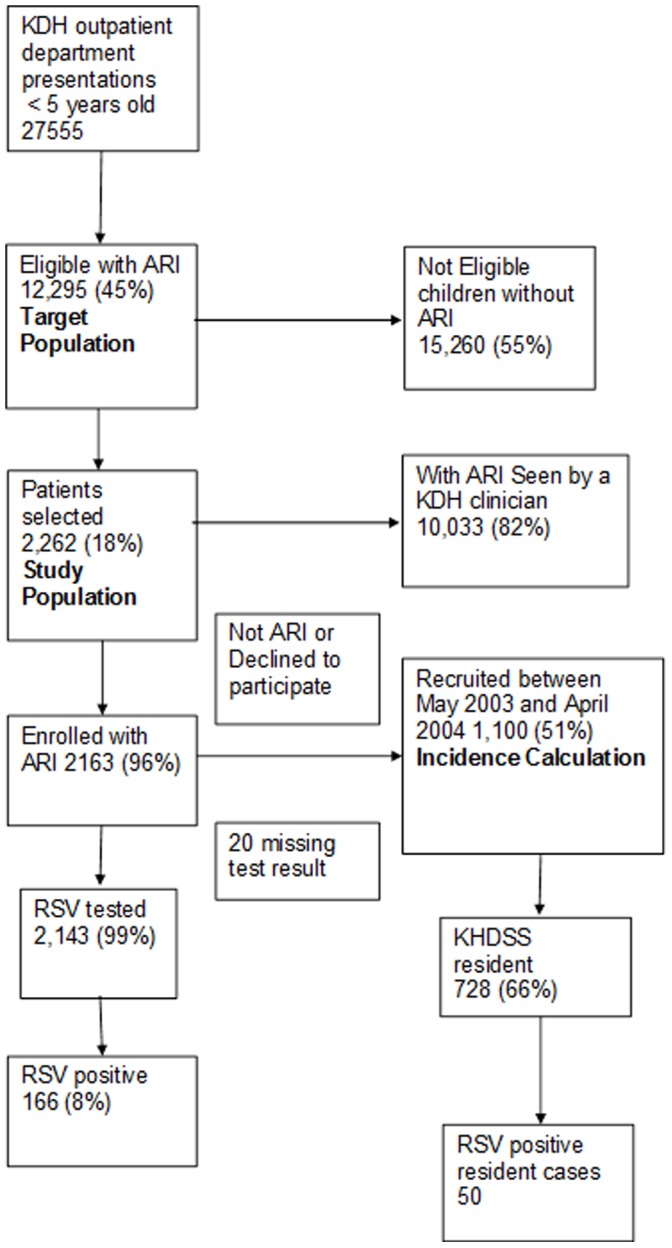
Flow diagram of recruitment and sample testing of children aged under 5 years presenting to Kilifi District Hospital from May 2002 to April 2004.

Between May 1, 2002 and April 30, 2004, 27,555 child <5yrs visits were made to the outpatient department (Fig. 1); 11,866 (43%) visits were made by infants and 14,498 (53%) were made by males. Of the total, 12,295 (45%) had a diagnosis of ARI (by Government Clinical Officers or as identified by study field worker based on a standard basic set of minimal symptoms) and were eligible for recruitment, 140 (0.5%) had no diagnosis record. Of those eligible 2262 (18%) were selected, 732 in 2002, 1101 in 2003 and 429 in 2004, and of these 99 (4%) either subsequently did not have a diagnosis of ARI when reviewed by a research clinician or had a missing diagnosis, and were excluded from further analysis. The remaining 2163 children had a nasal wash sample taken and tested for RSV antigen, but 20 (0.9%) children had a missing laboratory result.

A comparison of the characteristics of the study population and the target population is presented in Table 1. The proportion of infants in the study population was higher than in the population of children not selected for the study (44% vs 36%, P<0.0005), in particular due to differences in children aged 6–11 months and 36 months and over. The proportion of ARI presentations classified as LRTI within the study population of children (32%) was found to be significantly higher than the proportion with LRTI in the target populations (19%), even when adjusted for age (adjusted OR = 2.34 (2.10–2.60); P<0.001).

**Table 1:** Characteristics of ARI Study Population.

	Target Population		Study Population			Population Not Selected		P value(Fishers exact test)
	N	Percent	N	Percent	N		Percent	
**Diagnosis of ARI**								
URTI	9948	80.9	1479	68.4	8469		83.6	<0.0005
LRTI	2347	19.1	684	31.6	1663		16.4	
**sex**								
female	5919	48.2	1069	49.4	4850		47.9	0.195
male	6372	51.8	1094	50.6	5278		52.1	
**Diagnosis of malaria**								
no malaria	7682	62.5	1739	80.4	5943		58.7	<0.0005
malaria	4613∧	37.5	424*	19.6	4189		41.3	
**Age class of cases**								
<6 m	2505	20.4	490	22.6	2015		19.9	<0.0005$
6–11 m	2079	16.9	469	21.7	1610		15.9	
12–23 m	2903	23.6	549	25.4	2354		23.2	
24–35 m	2037	16.6	321	14.9	1716		16.9	
36–59 m	2771	22.5	334	15.4	2437		24.1	
**Infant**								
>365days	7711	62.7	1204	55.7	6507		64.2	<0.0005
< = 365days	4584	37.3	959	44.3	3625		35.8	

Notes.

* Diagnosis of malaria based on slide positivity.

∧ Clinical diagnosis of malaria.

$ Pearsons chi squared test comparing age in 5 strata.

### Epidemiology

Of the 2143 children who had an RSV test result, 166 (7.7%) children were positive. RSV positivity was not significantly associated with the sex of the child, even though there was a higher proportion of cases who were boys in the age group 0–5 months (30% vs. 23%). The prevalence of RSV in children with URTI was 6% (n = 80) and in children with LRTI it was 12% (n = 86) (exact; P<0.001). Of all RSV-associated cases 45 (27%) were aged 0–5 months and 36 (22%) were aged 6–11 months.

Parts of three RSV epidemics were observed with periods of approximately 8 months between the first and second epidemic and 15 months between the second and third epidemic. Seasonal variation in RSV cases was generally synchronized with total outpatient department presentations but was not temporally related with any meteorological measure which was coincident with inpatient population; where by the seasonal RSV pattern did not coincide with any obvious climatic signal.

### Clinical Characteristics

Of the 166 RSV-positive outpatient presentations in the study population, 113 (68.1%) had an URTI; 26 (15.7%) had LRTI, and 27 (16.3%) had severe LRTI. None of these children was referred for admission. LRTI was diagnosed in 23% of children under 5 years of age and in 20% of infants, of which an estimated 11% and 15%, respectively, were associated with an RSV infection. The distribution of RSV-associated LRTI by age and severity is presented in Table 2. Among cases of RSV disease the severity of the clinical spectrum declined with age: URTI was observed more commonly with increasing age and severe LRTI less commonly (29% in children <6 month to 8% in children 24 months of age and older).

**Table 2:** Age-distribution of RSV associated LRTI OPD presentations to KDH stratified by severity category in the Study Population.

	URTI			LRTI		Severe LRTI		
Age class	Positive	% in age class	Positive		% in age class	Positive	% in age class	Total Positive
<6 m	25	55.6	7		15.6	13	28.9	45
6–11 m	28	77.8	3		8.3	5	13.9	36
12–23 m	28	60.9	12		26.1	6	13.0	46
24–59 m	32	82.1	4		10.3	3	7.7	39
**Total**	**113**	**68.1**	**26**		**15.7**	**27**	**16.3**	**166**

The presenting signs of all enrolled ARI presentations are summarized in Table 3. In the sample of children the most frequent clinical findings were fever (38%) and crackles (27%) cough (25%) and fast breathing for age (20%). As shown in the Table, amongst those in which RSV was detected compared with those in which it was not, there were significantly more cases with crackles, nasal flaring, in-drawing, fast breathing for age and with fever. We assessed the differences in signs of RSV bronchiolitis (e.g. crackles, nasal flaring, wheezing) after stratifying on severity indicated by the presence of in-drawing and oxygen saturation (hypoxia) (Table 4). There were only 4 children with hypoxia who were also RSV positive too few to test for interaction. The interaction between in-drawing and crackles on RSV positive was found to be significant. In children with in-drawing the proportion with RSV infection was similar in children with crackles and those without crackles but in children without in-drawing, the proportion with RSV was almost double in those with crackles compared to those without crackles. There was a less significant interaction (although larger effect) between in-drawing and nasal flaring. Concurrent malaria parasitaemia and a clinical diagnosis of malaria were both significantly less common among RSV-positive children.

**Table 3:** Prevalence of clinical features in RSV positive and RSV negative children under 5 years of age presenting to OPD of Kilifi District Hospital between May 2002 and April 2004.

Signs		% RSV positive	% RSV negative	P	RR$
		n = 166	n = 1977		
Clinical∧	crackles	42.2	25.2	<0.001	1.63 (1.34–1.97)
	wheezing	4.8	2.3	0.065	2.13 (1.01–4.50)
	nasal flaring	6.6	2.3	0.003	2.66 (1.40–5.04)
	indrawing	13.9	5.7	<0.001	2.24 (1.47–3.40)
	hypoxia	2.4 (165)*	2.4 (1938)*	1.000	1.00 (0.37 2.74)
	fast breathing	26.8 (164)	19.2 (1963)	0.018	1.34 (1.03–1.75)
	cough	33.7	24.7	0.008	1.42 (1.13–1.78)
	fever (>37.5)	54.8	36.4 (1962)*	<0.001	1.54 (1.33–1.80)
Concurrent illness	Slide positive	14.6 (75)*	34.4 (850)*	<0.001	
	Clinical diagnosis of malaria	10.2	20.4	0.001	0.55 (0.35–0.87)

**Footnotes.**

$ MH RR risk adjusted for ageclass.

* Number of study participants included in this estimate.

∧ prostration was very rare and is not included in the table.

**Table 4:** Relationship between disease severity and clinical features in RSV positive and RSV negative children under 5 years of age presenting to OPD of Kilifi District Hospital between May 2002 and April 2004.

Signs		Indrawing			No indrawing				
		% RSV positive n = 23	% RSV negative n = 113	RR P value	% RSV positive n = 143	% RSV negative n = 1864	RR P value	Crude RR	RR$
**Clinical^∧^**	Crackles	78.3	73.5	1.07(0.84–1.36)0.6306	36.4	22.3	1.63(1.29–2.06)0.0001	1.67(1.38–2.03)	1.45(1.21–1.73)*
	Wheezing	13.0	11.5	1.13(0.35–3.66)0.8346	3.5	1.8	1.97(0.78–4.98)0.1444	2.07(0.99–4.31)	1.57(0.76–3.22)
	nasal flaring	30.4	27.4	1.11(0.56–2.20)0.7700	2.7	0.8	3.48(1.17–10.34)0.0177	2.85(1.50–5.39	1.51(0.86–2.65)
	fast breathing	73.9	58.0	1.27(0.95–1.70)0.1555	19.1	16.8	1.14(0.80–1.62)0.4741	1.40(1.07–1.83)	1.18(0.92–1.52)

**Footnotes.**

$ Adjusted for indrawing.

* Significantly different.

### Incidence of OPD Attendance

The population of some 240,000 individuals has been under demographic surveillance from year 2000 as part of the KHDSS. Over the 1 year period between May 2003 and April 2004 for which incidence was calculated, the number of outpatient cases recruited was 1100 children 728 of whom were KHDSS residents and 331 of these aged <1 year. Of the 81 cases of RSV identified during this period 50 cases were residents 23 of whom were infants. Among the 331 outpatient infants resident in KHDSS 17 (5%) had a positive RSV test and an URTI and 6 (2%) had a positive RSV test and a LRTI. Similarly, in resident children under 5 years the prevalence of RSV in ARI cases was 6.9% (50/728), in URTI cases was 5.2% (28/538) and in LRTI was 11.6% (22/190). The estimated age-standardized population based incidence of ARI in children <1 year of age, 1–4 years of age and <5 years of age was 20473, 8867 and 11,172 cases per 100,000 population per year, respectively (Table 5). Correspondingly, the LRTI incidence rates for children <1 year of age, 1–4 years of age and <5 years of age were 6185, 2010 and 2443 cases per 100,000 per year, respectively. The population based incidence rates for RSV-associated ARI were 1423, 603 and 767 per 100,000 per year for children aged <1 year, 1–4 years and <5 years, respectively. The incidence per 100,000 of RSV associated LRTI in children aged <1 year, 1–4 years and <5 years was 557, 290 and 283 per year respectively.

**Table 5:** Estimated age-standardized population based incidence of ARI in children per 100,000 population per year.

	ARI	LRTI	RSV-ARI	RSV-LRTI
**<1 year**	20,473(19,523–21,469)	6,185(5,635–6,789)	1,423(1,186–1,707)	557(408–759)
**1–4 years**	8,867(8,550–9,296)	2,010(1,872–2,159)	603(525–692)	290(240–350)
**<5 years**	11,172(10,854–11,499)	2,443(2,308–2,588)	767(653–901)	283(254–316)

## Discussion

The features of symptomatic RSV infection in outpatient presentations and denominator-based incidence estimates are insufficiently described in sub-Saharan Africa. Our findings based on surveillance of outpatient presentations at the rural district hospital in Kilifi, Kenya over the period 2002 to 2004 provide an assessment of the community health burden and clinical characteristics of RSV cases attending an outpatient health facility. The study estimates that RSV accounts for 7.7% of all ARI presentations in children aged <5 years. Furthermore, ARI outpatient presentations account for 45% of presentations in the under fives. These data demonstrate the considerable burden contributed by ARI and RSV associated ARI to the health resources.

Extrapolation from our sample to the hospital population yields incidence estimates of RSV associated ARI of 1423 and 603 per 100,000 infants and children 1–4 years per year, respectively. The corresponding incidence of LRTI attributable to RSV was 557 and 290 per 100,000 infants and children aged 1–4 years per year, respectively. These estimates are substantially lower than reported for a birth cohort in the same area [10], [14]. In that study the incidence of RSV associated ARI was 34,500 cases per 100,000 child years of observation (CYO) while the incidence of RSV-LRTI cases was 9,000 and 10,400 per 100,000 CYO in all birth cohort children (median age 25 months) and infants, respectively. These two studies used the same case definitions and methods of sample collection and RSV diagnosis. However, the higher incidence estimates for the birth cohort study may be attributed to the active case detection (weekly during RSV epidemics) in place and enhanced self-referral to the study outpatient clinic between active home visits for which free treatment and travel cost was offered. The difference in incidence of ARI and RSV-associated ARI identified between passive surveillance of the present study and heightened case ascertainment of the birth cohort, is illustrative of the true and frequently unrecognised burden of RSV in the community.

One study from a tropical sub-Saharan African country, Mozambique [27], carried out over the main months of a single RSV epidemic (Feb-May 2000), reported a prevalence of RSV in outpatient infants of 7% in those presenting with URTI and 10% in those presenting with LRTI. These results accord well with the findings from the present study, where the equivalent proportions were 6% in infants with URTI and 12% in those with LRTI. Comparatively, studies from industrialised countries suggest a higher proportion of ARI outpatient department presentations are RSV positive [12], which may well be attributable to ease of access and differential health service utilisation behaviour.

One in every six of the RSV positive children presenting to the KDH outpatient department had syndromically defined severe or very severe LRTI. Although eligible for hospital admission according to IMCI guidelines [28] none of the 27 cases were referred for admission. A similar observation was reported in a birth cohort study within the same population and reinforces the warning that hospital data will under-estimate the burden of RSV associated severe LRTI [10], [14]. However, since the well trained clinicians who reviewed these children did not consider them sufficiently unwell to refer for admission the result supports the assertion of definitions which have low specificity for LRTI [29] RSV associated disease in outpatient presentations appears to share similar features typical of those RSV cases seen in the hospital in-patient wards. The clinical signs crackles, nasal flaring, fast breathing, fever and lower chest-wall in-drawing were all associated with RSV similar to reports of the inpatient cases from the same population [15] as in other populations [27]. These signs were present at a lower percentage than those in a study from Mozambique [27]. Almost half of the outpatients with RSV associated ARI had crackles and were febrile and a higher proportion of RSV cases relative to those without a diagnosis of RSV were associated with more severe disease (16.3% vs 7.7%). Stratification on severity indicated that RSV infection in non-severe cases (without in-drawing) was associated with increased incidence of crackles and nasal flaring. The prevalence of malaria (clinical and slide confirmed) was lower in those children with an RSV infection in this study population than in those children without an RSV infection.

While RSV associated severe LRTI in outpatient cases was, like IP cases [15], most common in early infants (<6 m of age), overall there were as many cases in children aged one year and over as in infants. Clearly, maternal vaccination to boost infant passive immunity or vaccination to directly protect early infants would be beneficial in preventing the most severe RSV. However, delayed vaccination to the second 6months of life where immunogeniocity and tolerance are much improved would potentially have high benefit by preventing much of outpatient RSV burden.

There are some limitations to our study. First, passive surveillance at health facilities that depend mainly on the study population actually using hospitals or health centres may contribute to difference between these and other studies. Incidence rates for admissions were double in the region immediately surrounding the hospital [15] which would suggest that individuals may opt to go to Health Facilities closer to their places of residence. While Kilifi District Hospital is the main admitting facility in the KHDSS, there are several Health centres that patients can visit to access outpatient care. This implies that the catchment populations for outpatient services versus inpatient services may not be comparable. Secondly, there is reduced sensitive of case surveillance because of the viral detection method used. Using RT-PCR methods and serologic examination affords greater sensitivity [14], [30], [31], [32]. However it important to note that these are not routinely available in resource limited settings. Thirdly, it has been reported that HIV has some influence on the RSV infection [33]. This study did not investigate the influence of HIV on the study population thus the influence of HIV as a confounder is not precisely known. However, the overall HIV-1 prevalence in women attending to KDH antenatal care in 2004 was 4.8% (95% CI 3.4–6.6) [17]. Lastly, only a sub sample of outpatient presentations were included which is not optimal. Several adjustments were undertaken to account for this. Mainly in order to standardise LRTI diagnosis for the purpose of incidence estimation, the prevalence of LRTI in the KDH outpatient department population was imputed using the study LRTI diagnosis. The assumption is that the diagnosis of LRTI in the study (based on WHO criteria) is more standard and reliable than that in the KDH outpatient department. The different diagnostic criteria of ARI applied within the study and by Government clinicians could potential influence our results. However, because we wanted to capture all respiratory infections we used minimal symptoms to define a respiratory infection in the study population. No adjustment was done for ARI because we do not think there was any ambiguity about the diagnosis of an ARI in this population. Additionally, overall incidence estimates of LRTI and RSV-LRTI for children aged <5 years were adjusted for the differences in infant population between the study sample and the KDH outpatient department presentations. Importantly, as described recruitment was done in a random manner and a comparison between the whole population and of the sample indicates that the characteristics were largely similar. Nevertheless it is acknowledged that the study sample may not be truly representative of the population as a whole.

The burden of RSV in Africa is not fully appreciated by most public health officials given that RSV, albeit an important culprit [10], [34], [35], is not the only cause of pneumonia in African children and is not as widely exposed and more specifically because it is only one of countless other pathogens that characteristically cause respiratory disease in African children. These data are useful principally because one, as the data show not all serious cases get to hospital; an issue that is of particular concern in developing countries where a minority of cases actually make it to hospital [14], [36] and two because in general, inpatient hospital studies ignore the individually less serious, but still important, disease burden in the community. In summary, this is one of few studies of RSV in outpatient populations in Africa. On the basis of our findings we estimate that in children less than 5 years RSV infection results in approximately 3% of all outpatient presentations and 8% of all presentation with a diagnosis of ARI. In comparison, a clinical diagnosis of malaria is made in 36% of all sick child visits roughly half of which will be true malaria events based on a positive blood film. However, infection with RSV was associated with more severe disease thus its contribution to disease is significant.
